# Spatial Variation in Body Condition of a Coastal Sentinel, the Little Penguin, Reflects Marine and Terrestrial Factors

**DOI:** 10.1002/ece3.71361

**Published:** 2025-05-06

**Authors:** Melanie R. Wells, Annie Philips, Mary‐Anne Lea, Scott Carver

**Affiliations:** ^1^ Department of Biological Sciences University of Tasmania Hobart Tasmania Australia; ^2^ Institute for Marine and Antarctic Studies Battery Point Tasmania Australia; ^3^ Wildlife Veterinary Consultant Hobart Tasmania Australia; ^4^ Centre for Marine Socioecology University of Tasmania Hobart Tasmania Australia; ^5^ Odum School of Ecology University of Georgia Georgia USA; ^6^ Center for the Ecology of Infectious Diseases University of Georgia Georgia USA

**Keywords:** anthropogenic stress, habitat loss, marine productivity, parasitism, seabirds

## Abstract

Seabirds are widely considered sentinels of their coastal ecosystems. However, biological associations between specific marine and terrestrial factors and seabird health are less well understood. Here, we investigate the associations between habitat‐scale processes and variability in body condition of breeding little penguins *Eudyptula minor,* across 15 populations in Lutruwita/Tasmania, Australia. Sites represent a range of nesting habitats, oceanographic conditions and localised anthropogenic disturbance, providing an unparalleled opportunity to assess the impact of multiple processes shaping health. We show significant regional differences in body condition, with eastern populations consistently in better body condition than northern. Using Bayesian multilevel models, we found marine productivity to have the strongest relationship (coef. 0.52, 95% CI [0.13, 0.91]). Body condition was also negatively related to habitat loss, expressed as total road length around the colony, and ectoparasite load, with parasitism having a greater negative association in females. By assessing health across a spatially robust number of populations, rarely performed in seabirds, we reveal marine, terrestrial and sex relationships, indicating potentially important processes shaping health that would otherwise be difficult to discern.

## Graphical abstract

We detected spatial heterogeneity in the early breeding body condition of a coastal resident, the little penguin. Using Bayesian hierarchical modelling, we looked at several different environmental variables and their associations with conditions. We found differing sex‐based associations and that local marine productivity had the strongest positive association with condition, but urbanisation and parasite load were negatively associated with condition.

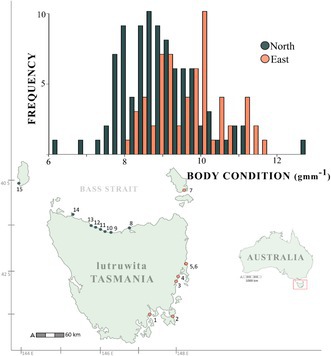

## 1 Introduction

Seabirds are widely regarded as sentinels for marine and coastal ecosystems (Piatt et al. 2007; Velarde et al. 2019). Through top‐down trophic interactions, they can be used as biomonitors of oceanographic and climate processes (Jones et al. 2018; Piatt et al. 2020). Owing to their reliance on terrestrial ecosystems for breeding and moulting, seabirds are also affected by terrestrial factors such as climate events (Quintana et al. 2022), habitat change (Kavelaars et al. 2020) or other forms of human‐induced disturbance (Watson et al. 2014). Physiological aspects of seabird health, such as body condition, stress or parasitism, can be used as indicators of marine and terrestrial factors (Megía‐Palma et al. 2022; Rakotoniaina et al. 2016). However, assessing the contributions of individual processes integrated across these different ecosystems can be challenging. Therefore, knowledge regarding the extent to which seabird health is representative of specific processes from these different environments is often convoluted.

Connecting seabird health to marine and terrestrial factors has challenges; for example, many species are migratory and forage over great distances, so the degree to which an individual's physiological condition can be directly related to specific marine conditions can be difficult to ascertain (Brisson‐Curadeau et al. 2017). Furthermore, studies that have attempted to examine the extent to which metrics of seabird health are related to their environments often only gather findings from one or two locations, resulting in a potential geographical bias. For example, the assessment of seabird stress levels, attributed to anthropogenic causes, from only two populations may elicit bias due to spatial differences in environmental conditions or the inherent levels of human exposure in each population (Barbosa et al. 2013; Soldatini et al. 2015). While these studies are valuable in progressing knowledge of seabird stressors, they present a challenge when inferring and disentangling the multiple processes shaping health proxies.

Little penguins (*Eudyptula minor*) are an ideal species to examine the interconnectedness of seabird health to ecosystem factors (Evans et al. 2019; Finger et al. 2015). As the world's smallest penguin, weighing only *c. 1100* g (Chiaradia et al. 2016), this resident species has a limited foraging range (Hoskins et al. 2008). During breeding, individuals are constrained in their habitat use, as central place foragers (Phillips et al. 2019). However, even during nonbreeding periods, individuals mostly forage locally with short trips (McCutcheon et al. 2011), indicating year‐round prey availability and use of their terrestrial breeding and coastal foraging habitats (Gormley and Dann 2009). Due to the physical constraints around their foraging ability, they need to demonstrate flexibility in their behaviours (Cavallo et al. 2020). This adaptability extends to the diversity of physical characteristics of the nesting habitats they can occupy (Bull 2000). This means they can be sampled across spatial–environmental gradients to help disentangle processes associated with biological parameters. Distributed across southern Australia and parts of Aotearoa/New Zealand, they are listed as ‘least concern’ (IUCN 2020). However, localised declines are reported across their entire range (Bool et al. 2007; Cannell et al. 2016; Priddel et al. 2008; Stevenson and Woehler 2007). Moreover, as one of the few seabird species successfully persisting along the coastal human‐wildlife interface, they are an ideal sentinel species for exploring natural and anthropogenic factors in coastal marine ecosystems (Lundbäck et al. 2021).

Body condition is often used as a primary indication of health (Bateman et al. 2023; Boveng et al. 2020). Quick and easy to obtain, it offers insights into an individual's fat reserves and their ability to respond to acute threats (Sebastiano et al. 2019). For seabirds, declines in body condition have been correlated with parasitism (Sanz‐Aguilar et al. 2020), reduced immunocompetence (Tella et al. 2001), habitat loss (Burton et al. 2006) and pollutant load (Eckbo et al. 2019). Here, we hypothesise that seabird health is influenced by multifactorial marine and terrestrial processes and use body condition of breeding little penguins, hereafter referred to as LP, as a key health metric to test this. Specifically, we seek to address the importance of marine productivity, anthropogenic disturbance, breeding habitat loss, nest site characteristics and parasite load as possible determinants of body condition. We examine the relative associations of a suite of variables, some known to directly affect conditions such as marine productivity (Berlincourt and Arnould 2015) or parasitism (Hervías et al. 2013) and some which are completely exploratory in nature, such as relative urbanisation. We explore variation in breeding body conditions across 15 colonies in Lutruwita/Tasmania, Australia, across a spectrum of environments. We show distinct sex and spatial patterns in conditions, related to both marine and terrestrial factors, indicating that this metric of LP health appears to be reflective of these environments and highlights some key processes associated with the detected variability.

## 2 Materials and Methods

### 2.1 Body Condition

In all species, body condition acts as a primary indicator of an individual's health and fitness (Freeman et al. 2020). A decrease in body condition is typically the first negative consequence of a perturbance to health, and is a widely used metric of health in wildlife studies (Kophamel et al. 2021). Some seabirds will forgo a breeding attempt if a minimum condition has not been achieved during the prebreeding period (Chastel et al. 1995). In addition to acting as a catalyst for successful breeding, being in good body condition can buffer an individual's susceptibility to succumbing to disease, or increase the chances of survival in already diseased individuals (Sebastiano et al. 2019). A ratio index of body mass by body size is a rapid way of estimating an individual's fat reserves (Labocha et al. 2014). For LP, estimates of condition have been validated by dividing body mass (g) by flipper length (mm) from either the radial joint (Numata et al. 2000; Robinson et al. 2005) or ulna joint (Fahlman et al. 2006). In the current study, we took measurements from the ulna joint using callipers and mass was calculated to the nearest 10 g using a spring scale. As phenology is known to be associated with body condition, for example, during late chick rearing, breeding adults are generally in poor condition, compared to the prebreeding or moulting periods (Mortimer and Lill 2007); we have standardised our methodology by only sampling individuals in the early stages of breeding.

### 2.2 Study Sites

Lutruwita/Tasmania is considered a stronghold for LP and is thought to be home to over 60% of the Australian population (Dann et al. 1996). It is free from introduced European foxes ( *Vulpes vulpes* ), which have decimated colonies on the mainland (King et al. 2015; Kirkwood et al. 2014), so birds remain breeding in appreciable numbers along the coast and associated small islands, though this does not mean colonies are free from ecosystem‐scale threats. Populations are facing localised pressures from the growth of coastal urban development (Guy and Kirkpatrick 2018), offshore aquaculture expansion (Cullen‐Knox et al. 2021), pollutants (Wells et al. 2024) and pressure from domestic species such as cats ( *Felis catus* ) and dogs ( *Canis familiaris* ) also poses a great threat (Holderness‐Roddam and McQuillan 2014; Woehler et al. 2021). LP breed, rest and moult along a spectrum of anthropogenically altered environments, but little is known regarding local population ecology, and some populations have undergone declines and even extinctions in recent decades (Stevenson and Woehler 2007), demonstrating the susceptibility to threatening processes as yet unidentified.

**FIGURE 1:**
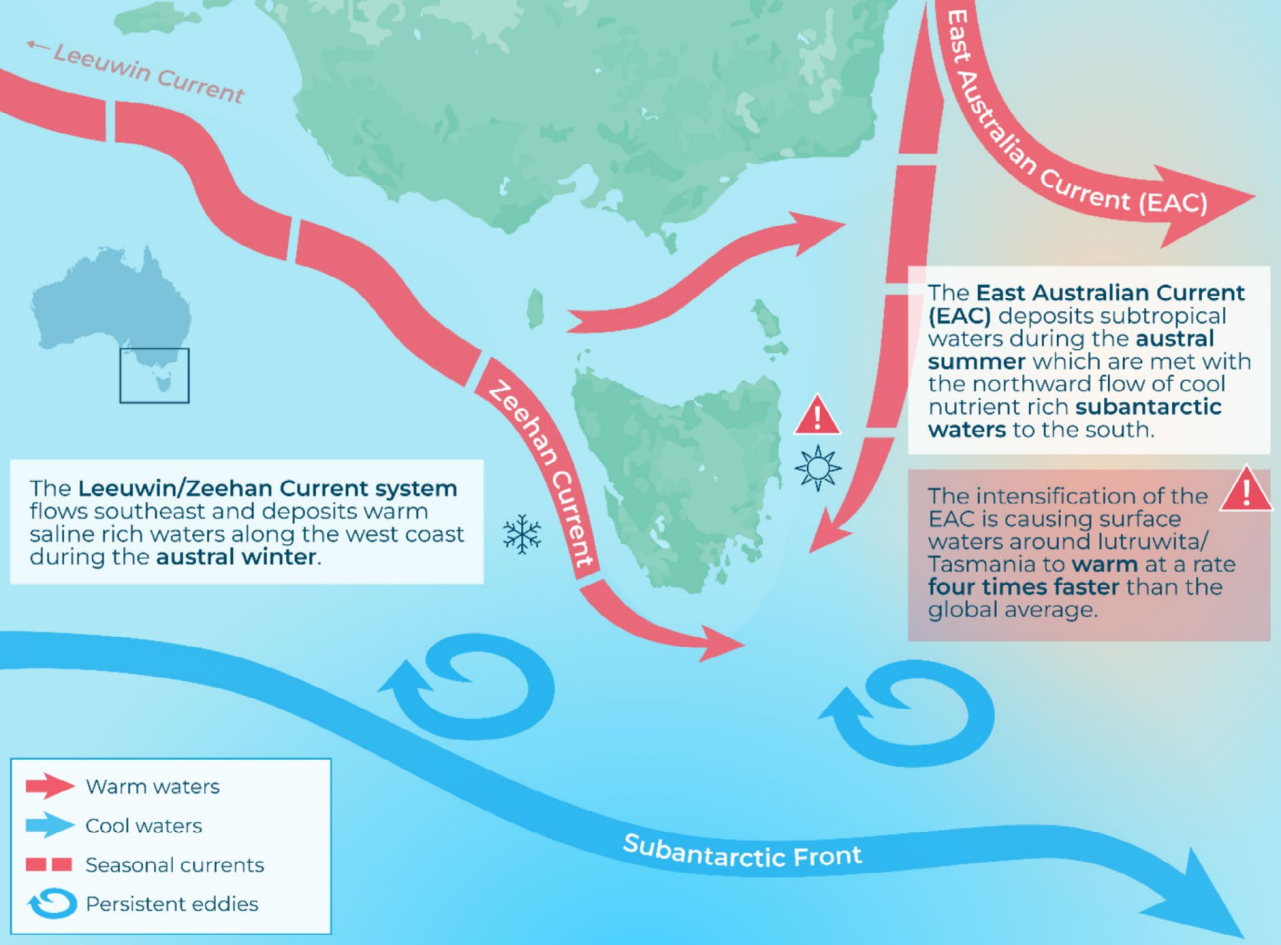
The oceanographic processes around Lutruwita/Tasmania as described in Duran et al. (2020) and illustrated by Stacey McCormack (Visual Knowledge Pty Ltd).

In this study, Lutruwita/Tasmania largely functions as a mesocosm, and the oceanographic regimes around its waters vary spatially. The Leeuwin/Zeehan current system flows southeast and deposits warm, saline‐rich waters along the west coast during the austral winter. The east coast is subject to the increasing southward extension and strengthening of the East Australian Current (EAC). The EAC deposits subtropical waters during the austral summer, which are met with the northward flow of cool, nutrient‐rich subantarctic waters to the south (Duran et al. 2020) (Figure 1). Due to the intensification of the EAC, surface waters around Lutruwita/Tasmania are warming at a rate four times quicker than the global average (Hobday and Lough 2011). Coupled with rapid climate change, this is giving rise to extreme marine heatwave events and leading to unprecedented disease outbreaks for aquaculture species (Oliver et al. 2017) which are projected to increase in both frequency and intensity (Oliver et al. 2018).

**FIGURE 2:**
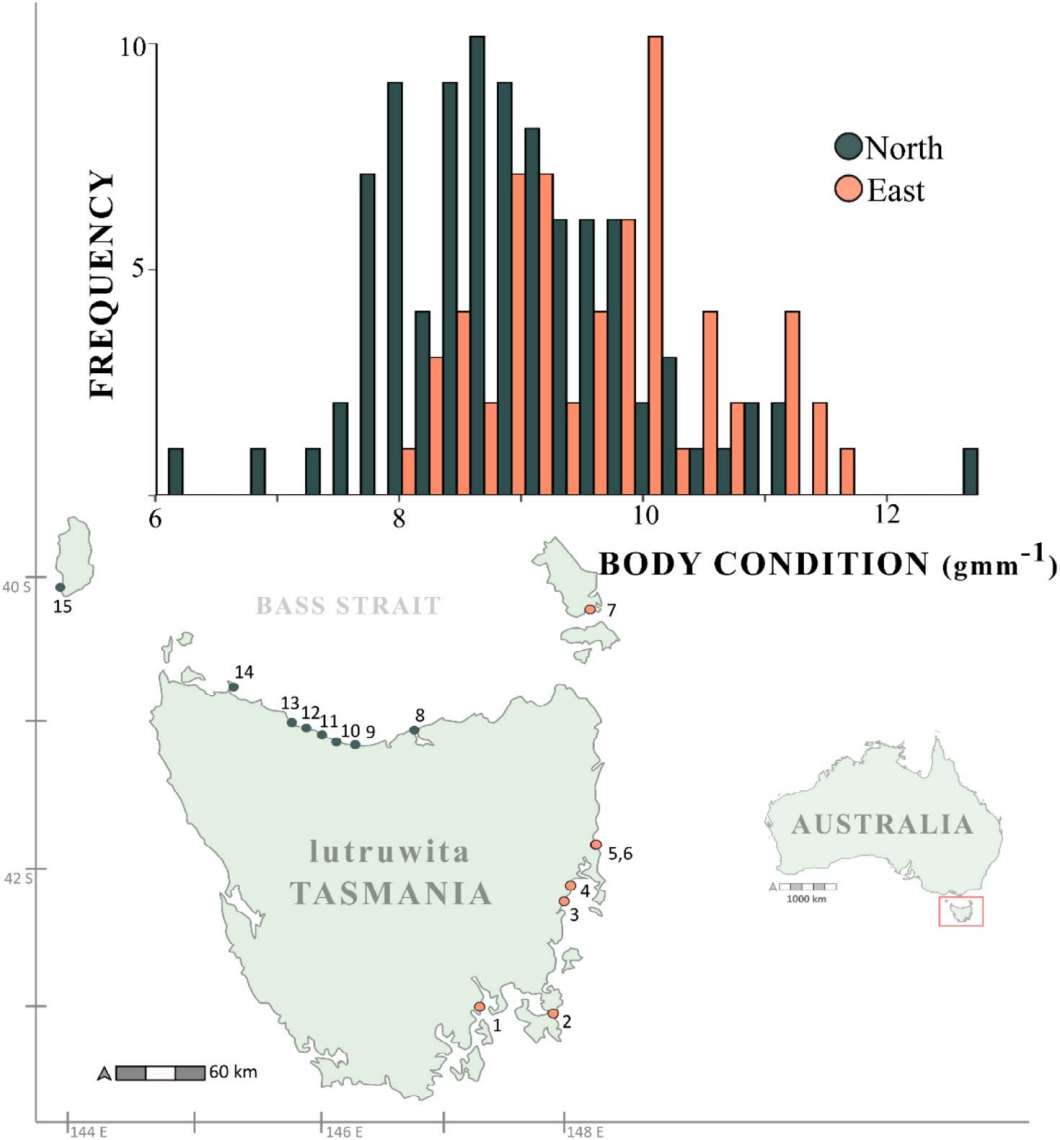
Histogram of little penguin body condition (gmm^−1^) around Lutruwita/Tasmania and study sites. North coast sites in blue and east coast sites in orange. Individual sites are represented by number; Derwent (1), Pirate's Bay (2), Little Christmas Island (3), Coswell (4), Bicheno (5), Diamond Island (6), Furneaux Islands (7), Low Head (8), Lillico (9), Ulverstone (10), Sulphur Creek (11), Burnie (12), Doctor's Rocks (13), Stanley (14) and King Island (15).

Sampling was conducted across 20 sites in total during the 2021–2022 breeding season. The sites represented a diversity of habitat types, oceanographic conditions and localised anthropogenic disturbance. These included nine island sites and 11 [Tasmanian] mainland sites. Data for two populations within the Derwent Estuary (Red Chapel and Boronia), two populations on King Island (Grassy and West) and three islands in the Furneaux Island Group (Fisher Island, Big Green Island and East Kangaroo Island) were pooled into three respective sites (Derwent, King and Furneaux) due to the proximity of the individual populations and low sample sizes, totalling 16 sites. The sites were broadly categorised as north or east coast, owing to their dominant oceanographic processes, with Furneaux Islands considered ‘east’ and King Island considered as ‘north’; additionally, no breeding birds were sampled at one site (Wedge Island), therefore body condition from breeding birds was compared across 15 sites (Figure 2).

### 2.3 Field Methods and Predictor Variables

Three sampling trips were conducted during daylight hours across breeding periods (Sep/Oct 2021, Nov/Dec 2021 and Jan/Feb 2022). During each trip, sites were surveyed by scouting suitable habitats for signs of nesting. Occupied nests were marked, and where possible, nests with known occupancy were selected for sampling. Ectoparasite load is known to be associated with variation in body condition (Hervías et al. 2013; Obendorf and McColl 1980). It was assessed by palpating and brushing the feathers and systematically inspecting typical areas of infestation (Espinaze et al. 2019). These were categorised as low, medium or high for ticks and fleas individually—two of the main types of visually conspicuous ectoparasites (Clarke and Kerry 2000). This assessment was always done by the same person, and broadly these categories were < 2 ectoparasites (low), 2–9 (medium) or 10+ (high). Birds were in early breeding stages, either incubating or brooding small chicks. General morphometric measurements were collected, and sex was determined based on bill measurements (Arnould et al. 2004); these data are presented in Appendix S5.

Due to the variation of physical characteristics of nests and the influence that nest type could have on fitness (Blay and Côté 2001; Sutherland et al. 2014) but unknown influence on condition specifically, the burrow structure (artificial or natural) was also recorded to explore any associations. Breeding density was recorded by counting the total number of active nests within a 10 m radius of the sampling nest (Brownlie et al. 2020) due to the association of increased competition (which density may be used as a proxy for) with penguin body condition (Tella et al. 2001).

To investigate the relationship of marine factors to LP body condition, which are widely used as indicators of prey presence and widely linked to condition (Ballard et al. 2010; Berlincourt and Arnould 2015), daily recordings of sea surface temperature (°C) and chlorophyll‐*a* concentration (mg m^−3^) (indicating primary productivity) were extracted using the *Raadtools* package in R 4.1.0 (R Core Development Team 2021; Sumner 2015). Sea surface temperature (SST) was derived from the Optimum Interpolation Sea Surface Temperature (OISST) v2 daily dataset (1/4° horizontal resolution) (Reynolds et al. 2007) and chlorophyll‐*a* (chla) data were derived from the Johnson et al. (2013) daily estimates (1/12° horizontal resolution). Data were extracted from within a 30 km zone around each colony, representing the approximate extent of foraging range during breeding, as recorded in other populations (Berlincourt and Arnould 2015; Hoskins et al. 2008; McCutcheon et al. 2011; Phillips et al. 2019). Daily SST and chla measurements were extracted and averaged across the 2‐month calendar period that each of the sampling trips fell in, broadly representing breeding in early spring, early summer and late summer.

To investigate associations between anthropogenic factors, known to negatively impact indices of penguin health (Ellenberg et al. 2013), two variables were used in an exploratory context for their associations with body condition specifically. Total length of roads within a 1 km zone of each colony was measured using the *units* and *sf* packages in R and the presence of industry (terrestrial and marine) within 10 km of each colony was recorded. Total road length is used as a proxy for intensity of localised human disturbance or urbanisation at the breeding site. The 1 km buffer zone represents the localised pressures around the nest site, as LP colonies in Lutruwita/Tasmania are generally geographically discrete, that is, they are physically restricted by sections of cleared land and, hence are quite small (Marker 2016). Industry presence represents both terrestrial‐based human disturbance and marine input of nitrogen or phosphorous through eutrophication or possible pollutants (Shtereva et al. 2015), hence the wider 10 km buffer to account for the greater range of LP in the marine environment. Both anthropogenic‐related variables are used in an exploratory sense, as their associations on condition, if any, are unknown. It may follow that increased urbanisation around a nest negatively associates with an individual's condition due to the constant stress and arousal associated with the cumulative factors inherent to a periurban population (Herbert et al. 2021). For the presence of industry, as a possible proxy for eutrophication, LP foraging effort has been linked to areas of increased nutrients (Phillips et al. 2019) and, hence may have a positive relationship with individual body condition. The variables are used as both a binary (industry presence) and continuous (road length) attempt at representing cumulative stressors associated with being an in‐shore forager that nest along a spectrum of anthropogenically disturbed habitats. All spatial land use data was obtained from publicly available data on ‘theLIST’ (Land Tasmania 2021) and visualised in QGIS 3.24.2 (QGIS Development Team 2023) (see Appendix S1, for site‐specific summary data of all parameters).

### 2.4 Statistical Analysis

**FIGURE 3:**
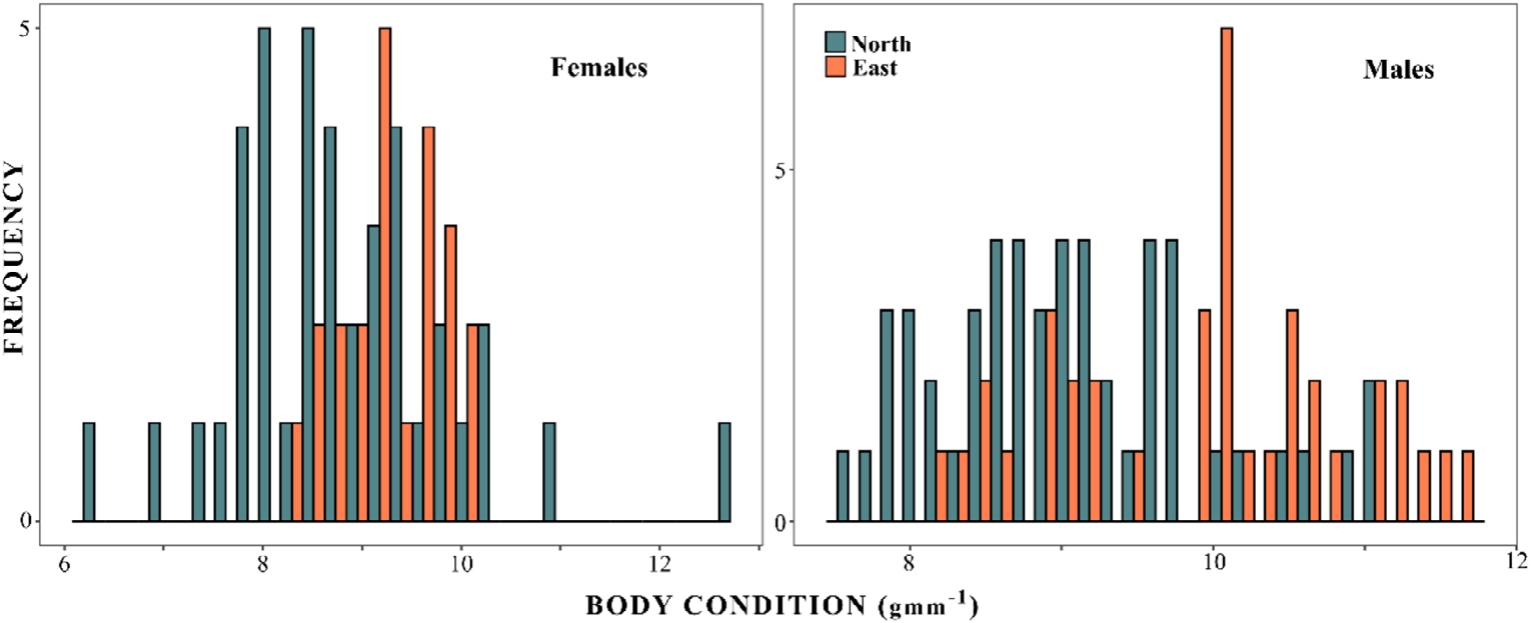
Histogram of breeding little penguin body condition (gmm^−1^) around Lutruwita/Tasmania for females (left) and males (right). North coast sites in blue and east coast sites in orange.

Analyses were conducted in the R statistical environment 4.1.0, using the Stan probabilistic language (Stan Development Team 2018) and the *brms* package (Bürkner 2017). We observed spatial variation in the pattern of body condition and sex‐specific differences (Figure 3). We report body condition data as means ± standard error of the mean (SEM). We then undertook analysis to ascertain the processes driving these patterns, electing to fit a single model consistent with recommendations from Sivula et al. (2020), as we did not have a distinct set of hypotheses among our variables from which to undertake model selection (Navarro 2019).

We investigated eight independent parameters, first visualising each variable by sex, which determined the inclusion of sex interaction terms. Independence between parameters was determined using a correlation matrix. Using a Bayesian multilevel model (BMLM) with sex as a fixed effect and site as a random effect, we included sampling period (nominal 1–3) which represents timing in the season, environmental variables (SST (°C) and chla (mg m^−3^) within 30 km of each site averaged across the 2‐month season for each sampling period), anthropogenic variables (cumulative road length within 1 km (continuous) and industry presence within 10 km (0/1)), burrow type (0/1), breeding density (continuous) and ectoparasite load (ordinal 0–6, preliminary analysis for flea and tick data showed comparable results so these data were pooled). Sex interaction terms were included for SST and burrow density due to apparent sex‐specific trends during data visualisation (Appendix S4). Numerical variables were *z*‐transformed to support comparability of posterior distributions and effect sizes.

Chlorophyll‐*a* data extracted from the shallow water Derwent Estuary site were comparatively much higher than values at other sites. Preliminary analysis omitting these sites produced comparable results; hence, data from this site were retained in subsequent analyses. The BMLM was conducted using a Gaussian distribution, and the *brms* default uninformative prior distributions. We ran 8000 Monte Carlo Markov chain (MCMC) iterations and a burn‐in of 6000 and assessed chain convergence with the Rhat (Gelman–Rubin) statistic by plotting the posterior distributions. The incorporation of a Gaussian process term to assess spatial independence of sites suggested a correlation between sites close together (coef. 0.33 ± 0.3, 95% CI [0.01, 1.26]), which decays with distance according to the length scale term, we elected to make spatial inferences based on the geographically broader regional scale (east and north). Posterior parameter estimates generated from the MCMC iterations were used to simulate 20,000 data points to further assess and visualise model adequacy (Appendix, S2). We report on the estimated mean of each parameter's posterior distribution as the coefficient, its standard deviation and 95% credible intervals (CI).

## 3 Results

Throughout the sampling period, we obtained body condition data from 151 early breeding stage LP across 15 sites. No ectoparasite estimates were obtained from 10 birds, and no burrow density data were obtained from an additional 18 individuals due to time constraints; therefore, 123 observations of breeding body condition were used in the model. Male breeders were in better overall condition (9.43 ± 0.11, range: 7.46–11.65 gmm^−1^) than females (8.95 ± 0.13 range: 6.24–12.65 gmm^−1^), and there was evidence for extrinsic variation in body condition between breeding sites around Lutruwita/Tasmania (ANOVA F_14,136_ = 3.645, *p* < 0.001). The largest differences were between Coswell and Burnie, 10.38 ± 0.27 and 8.53 ± 0.3 gmm^−1^, respectively (Appendix S3). Due to a lack of fine‐scale spatial independence between sites, we categorised them regionally, and populations were in better condition on the East Coast compared with northern populations (Figure 2). The spatial patterns in condition were greater than sex‐specific differences, with females from the east coast being in better average condition than males from the north coast, 9.39 ± 0.11 SEM gmm^−1^ and 8.99 ± 0.12 gmm^−1^, respectively (Figure 3).

**FIGURE 4:**
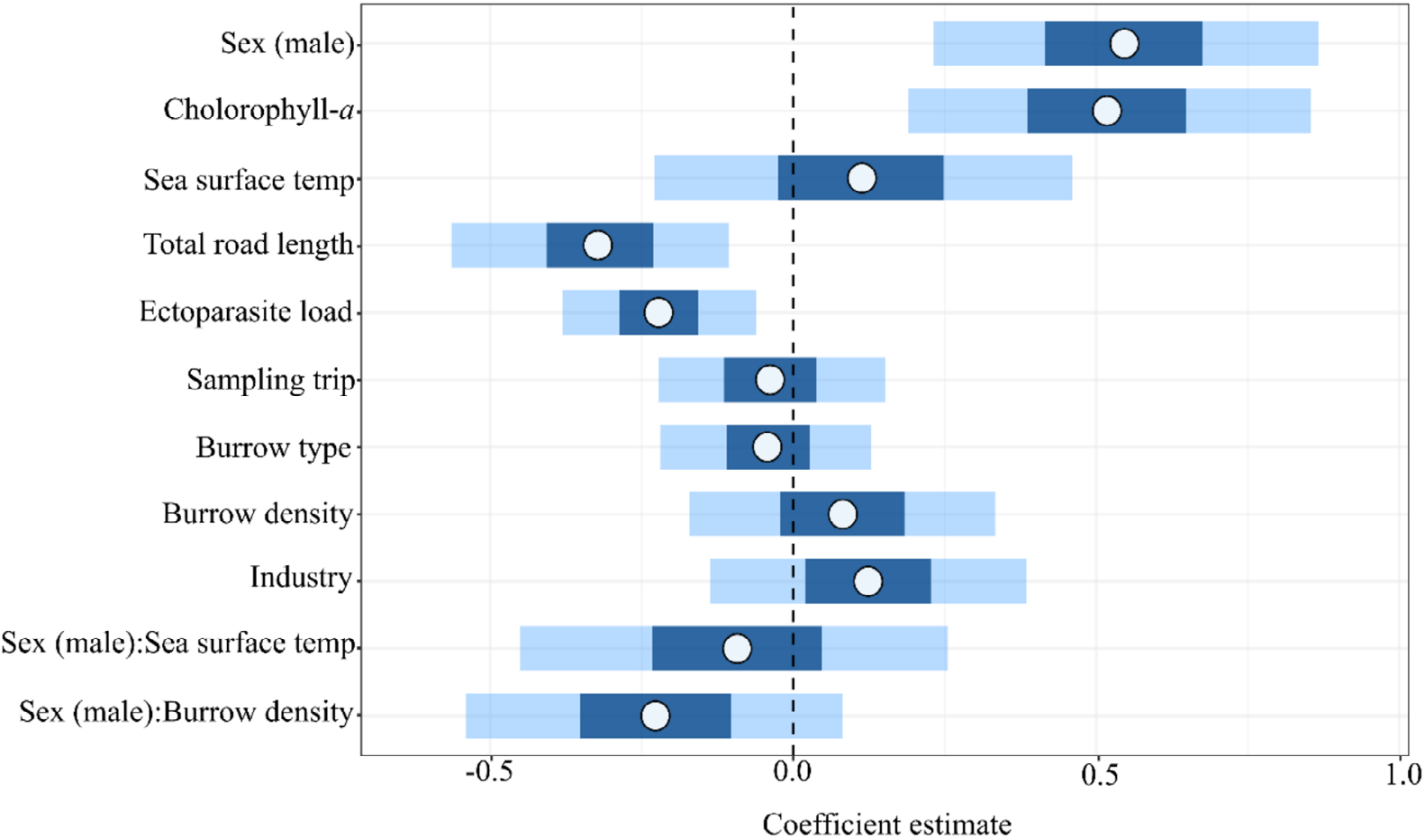
Coefficient estimates with 95% credible intervals of posterior distributions for parameters used in Bayesian multilevel model examining determinants of little penguin breeding body condition (*n = 123*). Parameters are sex (male plotted by default), chlorophyll‐*a* (mgm^−3^), sea surface temperature (°C), total road length within 1 km of site (km), ectoparasite load (0–6), sampling trip (1–4), burrow type (artificial/natural), burrow density (*n* ^−10m^), industry within 10 km (Y/N) and sex interactions with sea surface temperature and burrow density.

**FIGURE 5:**
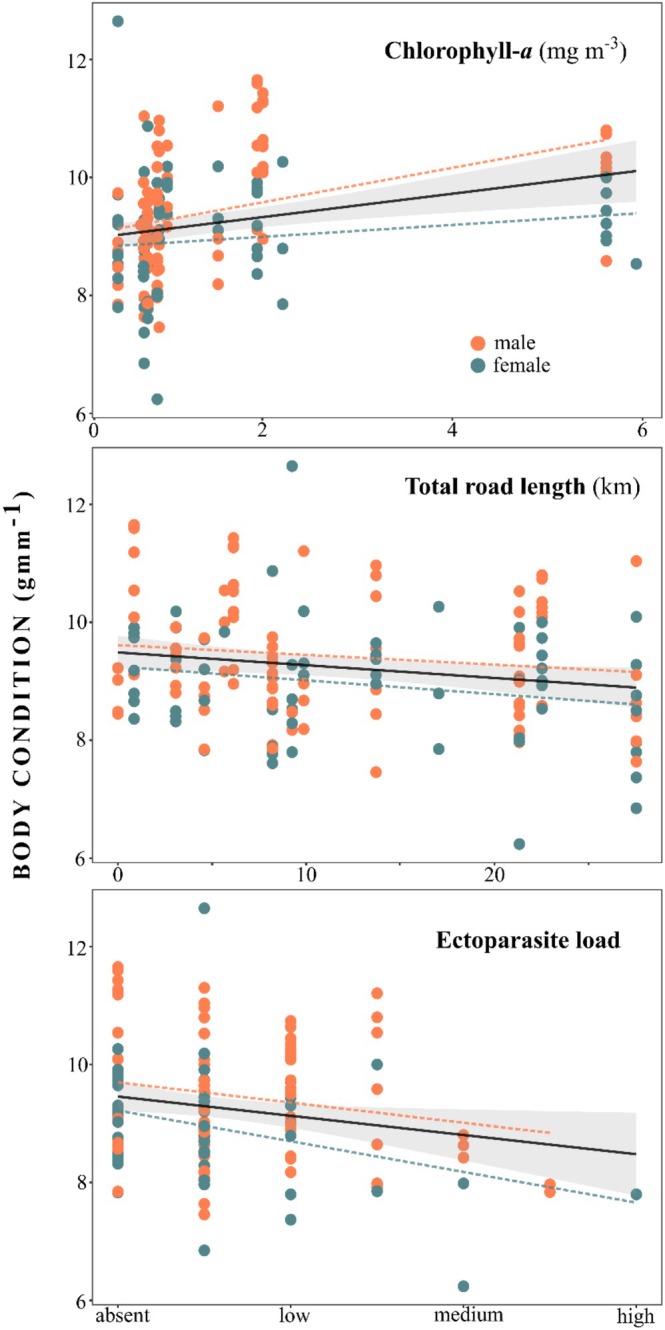
Significant processes determining little penguin breeding body condition, chlorophyll‐*a*, total road length within 1 Km of site and ectoparasite load. Black line represents regression coefficient with 95% confidence intervals and dashed orange line represents trend for males only and blue for females.

Chlorophyll‐*a* concentrations within 30 km of colonies were positively related to body condition (coef. 0.52 ± 0.20, 95% CI [0.13, 0.91]) and total road length within 1 km of the colony and ectoparasite load had negative associations with LP body condition (coef. −0.32 ± 0.14, 95% CI [−0.60, −0.06] and coef. −0.22 ± 0.10, 95% CI [−0.41, −0.03] respectively). Notably, ectoparasite load had a greater negative association with female condition (Figure 5). Although sex‐specific trends were apparent in both localised SST and burrow density, the 95% credible interval estimates of these variables and all other parameters, including the temporal variable pertaining to the timing of sampling, overlapped zero, indicating insignificant relationships with body condition (Figure 4).

## 4 Discussion

Seabirds are often labelled sentinels of the environments they rely on for breeding and foraging, but what specific environmental processes shape their health and fitness remains a knowledge gap. We considered a suite of both marine and terrestrial factors as possible determinants of LP body condition, across a broad spatial representation, to disentangle these interconnected processes. We have highlighted key regional differences in conditions around Lutruwita/Tasmania. Local marine productivity (chla as an indicator for prey availability) had the strongest relationship with condition, and variables related to their breeding sites (total road length within 1 km and ectoparasite load) were also important factors. Our findings suggest that the body condition of breeding LPs is associated with both marine and terrestrial factors and that there are sex‐specific differences in these relationships.

We reported generally better body condition in males than females, consistent with previous research for this species (Kato et al. 2008). Ecological niche theory has been linked to sexual size dimorphism in other penguin species, suggesting that inherent differences in foraging behaviours result in males accessing higher‐quality prey (Delord et al. 2021; Pichegru et al. 2013). Some sexual segregation in dive depth and trip duration has been reported for LP, although a high amount of intraspecific variability is also reported (Hoskins et al. 2008; Kato et al. 2008). Conversely, other studies have reported no sex differences in foraging strategies (Berlincourt and Arnould 2015; Camprasse et al. 2017; Pelletier et al. 2014). For central place foragers such as seabirds, differences in local marine conditions apparently have the greatest power for explaining differences in foraging strategies and specifically body conditions (Steinfurth et al. 2019). Marine variability is typically used as a proxy of prey availability (Evans et al. 2019) and fine‐scale environmental conditions have been demonstrated to influence the foraging behaviour of local LP populations (Phillips et al. 2019). In the absence of site‐specific foraging behaviour or diet data, we were unable to infer differences in local strategies; however, we have demonstrated that local marine conditions, specifically chla, can be an effective proxy for the likely body condition of breeding birds. The regional pattern we observed was greater than the sex differences, in contrast to previous findings from only one site, reporting that males are always heavier (Chiaradia et al. 2016). We found that females from the east coast were in better average condition compared with males from the north coast. However, it should be noted that the ratio index of flipper length by mass is one means of estimating conditions in lieu of fat mass validation data, and inherent stochastic variation in the size of individuals independent of sex differences will certainly exist. Size and condition data were explored in the Supporting Information, and although slight differences in the strength of the relationship between flipper and mass for each sex were detected, these were not significant.

Remotely sensed chla data is commonly used to represent surface layer marine productivity characterised by phytoplankton biomass, and has ecological significance, with peak breeding for LP coinciding with peak chla concentrations (Berlincourt and Arnould 2015). Using remotely sensed chla data, however, is challenging when extrapolating from coastal or shallow environments. The influence of suspended sediments in the water column can interfere with the spectral signature of chla (Green et al. 2000). The sites in the Derwent Estuary recorded the highest levels of chla, potentially indicating outliers in the data. Preliminary analysis omitting these sites, however, produced comparable results. The higher chla concentrations for this area are likely a result of increased nutrient mixing and persistent phytoplankton blooms in the waterbody, and are comparable with previous observations for this region (Wild‐Allen et al. 2013). Furthermore, one of the few previous studies on these populations examined foraging behaviour relative to localised fine‐scale marine conditions and found birds from the Derwent Estuary concentrated foraging effort in areas of increased nutrients, suggesting prey availability (Phillips et al. 2019).

Bass Strait, lying north of Lutruwita/Tasmania, is considered a region of low productivity, characterised by chla and nutrient data, with the major source of nutrients coming from the cold north‐flowing subantarctic waters. The stronger east coast influence of nutrient‐rich waters could explain why these regions support the acquisition of greater fat reserves for LP, compared to the north coast. Furthermore, due to the mixing of subtropical southward‐flowing waters from the EAC and subantarctic nutrient‐rich waters along the east coast, oceanographic trends for this region have indicated an increase in winter primary productivity. Availability of rich prey resources prior to the onset of breeding is critical for marine predators (Salton et al. 2015). It should be noted, however, that the cross‐sectional approach to the study design, maximising the number of study sites visited at a point in time, did not allow for examining individual foraging or breeding efforts, which may elicit inherent bias in the regional patterns observed. Furthermore, the phenological status of birds was broadly categorised; early‐stage breeders were used, but the fine‐scale phenological stage was not explicitly examined (i.e., incubation or brooding), and body condition has been shown to drastically fluctuate between these stages (Mortimer and Lill 2007), which was possibly evident with the broad range detected in female condition. Due to the asynchronous breeding of LP across the austral summer, we incorporated the seasonal information pertaining to the sampling trip (i.e., early spring, late spring or summer), which had a negligible association with condition. However, as we did not conduct any mark–recapture of individuals, it is unknown whether an individual sampled during summer was completing its first, second or possibly even third breeding attempt for the season (Joly et al. 2023), which would undoubtedly have a cumulative and unaccounted‐for negative impact on observable condition. As variation in chla concentrations has been consistently linked to timing of LP breeding (Joly et al. 2023), it would be worthwhile to build on these inferences with a longitudinal study where fine‐scale oceanography can be measured to validate our observed spatial patterns. Nonetheless, we suggest considering the East Coast as an area of possible greater prey resources for LP.

Marine productivity, however, is only one consideration, and although birds from the site with the highest chla concentrations (Derwent) were among the top five sites for good condition, they exist in heavily urbanised areas. These populations face pressures from human disturbances and pollution, with over 22 km of total road length within the 1 km buffer zone of the colony, second to Burnie on the north coast, where the remaining undeveloped coastline is habitat to LP that was in the poorest condition around Lutruwita/Tasmania. In addition to determining the importance of local marine productivity (measured by chla) as a determinant for good condition, this study has also highlighted key processes which may negatively affect the condition of breeding birds such as parasite burden and loss of breeding habitat.

We examined parameters representing human disturbance, and total road length within a 1 km zone of each colony, indicating the extent of localised breeding habitat degradation, had a clear negative relationship to conditions for both sexes. This suggests biologically negative consequences from habitat destruction, a seemingly intuitive finding. Habitat loss is a major cause of biodiversity decline (Pereira et al. 2010); moreover, it is a major cause of decline in seabirds (Croxall et al. 2012). For penguins specifically, terrestrial habitat loss has been linked to the introduction of alien species and associated predation or grazing pressures, and increased disturbance from human and infrastructure presence (Trathan et al. 2015). Burnie, the colony along the north coast where breeding birds were in the poorest condition, is subject to the greatest extent of habitat loss, as well as being adjacent to Tasmania's largest cargo port. We suggest that the cumulative stress associated with the multifactorial pressures of living along an urban fringe as a small flightless bird negatively affects condition, and investigations into markers for physiological stress may be warranted. Additionally, habitat loss and urbanisation can have complicated cascades on parasite–host dynamics (Delgado‐V and French 2012), with some studies describing positive relationships between urbanisation and parasite richness (Belo et al. 2011).

We have revealed an overall negative association between parasitism and LP condition. The ecological relationship between a parasite and its host is complicated. Generally, most ectoparasites can complete their life cycles with minimal consequences to a healthy host (Ferrer and Morandini 2019). Negative effects from parasitism on hosts may be more likely during the co‐occurrence of other stressors, which in turn may also affect conditions, such as introduced predators (Hervías et al. 2013), pollution (Blanar et al. 2009), habitat loss (Dharmarajan et al. 2021) or nutritional stress (Espinaze et al. 2020). This synergistic interplay of multiple pressures on an individual is more likely to reduce resilience and result in them succumbing to disease processes.

Prominently, parasitism had a greater negative association with female conditions. This could be because females begin the breeding season with the energetically costly phase of egg production and laying (Rebstock and Boersma 2018) or that due to the intrinsic sexual size dimorphism, females generally have less fat reserves and hence have less of a buffer to cope with the impact of parasites. The condition of female LP fluctuates substantially within a breeding season compared to males (Mortimer and Lill 2007). It is, therefore, likely that due to an inherent state of physiological stress, females may be more vulnerable to succumbing to negative impacts from parasitism or other stressors (Sanz‐Aguilar et al. 2020). This has been substantiated through female‐biased mortalities in studies investigating the cause of death in beach cast carcasses across the LP range (Dann 1992; Obendorf and McColl 1980). Moreover, female LP had significantly lower survival rates post‐rehabilitation after a considerable oiling event (Goldsworthy et al. 2000). Comparable female‐biased mortality rates have been documented in similarly sexually dimorphic African penguins ( *Spheniscus demersus* ) (Pichegru and Parsons 2014), possibly accelerating the rate of decline in small populations of this critically endangered species. This highlights the necessity of reporting sex differences during health investigations (Kophamel et al. 2021) to identify vulnerable demographics for targeted conservation management or intervention strategies, such as supplement feeding (Sebastiano et al. 2019) or parasite control (Alderman and Hobday 2017).

We have demonstrated that health, as represented by a body condition index, varies across the range of LPs and is possibly an important indicator of larger ecosystem processes. Longitudinal observations would be valuable to further corroborate these findings and substantiate the importance of marine productivity in years of variable productivity (Champagnon et al. 2018). Nonetheless, we have observed considerable spatial differences in the condition of breeding LP. Birds breeding on the east coast of Lutruwita/Tasmania were consistently larger and in better condition than those occurring along the north coast, irrespective of sex or timing in the breeding season. This extrinsic spatial variation in condition was most strongly related to chla (used as a proxy for marine productivity and hence probable food availability) and negatively related to the extent of breeding habitat loss and parasitism. Furthermore, we have highlighted that potential processes that negatively influence body condition, such as parasitism, have a greater negative relationship for females, who are often in comparatively poorer condition than males. This suggests potential greater vulnerability to other health perturbances in response to threatening processes.

Our findings indicated the strongest spatially variable determinant for condition in breeding LP was marine productivity (chla) and we highlight east coast populations. The upper threshold of this association, however, is unknown (Phillips et al. 2019). While this study is lacking in longitudinal data, it can be postulated that increases in the frequency and intensity of marine heatwave events may cause bottom‐up trophic cascades that will reduce predator fitness (Bost et al. 2015; Piatt et al. 2020). Although LP have plasticity in foraging strategies (Camprasse et al. 2017), they are still constrained by range. It is predicted that range‐restricted seabird populations are at greater risk of negative impacts from major marine disturbance events (Woehler and Hobday 2023). These events alter the entire prey field structure through the occurrence of harmful algal blooms causing mass mortalities or diseases in lower trophic level organisms (Smith et al. 2021). Cascading consequences from disease outbreaks in key prey species have previously culminated in mass mortalities of LP (Dann et al. 2000). As the waters off eastern Lutruwita/Tasmania are some of the fastest warming globally (Hobday and Lough 2011), marine heatwave events are predicted to increase (Oliver et al. 2018) and will likely result in reductions to success and survival for local LP populations.

The data here provide valuable cross‐sectional insights into individual and population‐level LP health, and fine‐scale multi‐site longitudinal investigations may continue to disentangle key processes shaping health. Nonetheless, this study demonstrates that measures of species health are not uniform across their distribution, and for a resident coastal seabird, can be associated with specific marine and terrestrial processes. We highlight negative associations between body condition and breeding habitat loss (total road length around colony) and parasitism, suggesting to managers that peri‐urban colonies may be at greater risk of other health perturbances. Employing a concurrent assessment of multiple populations, we highlight the need for a holistic perspective into factors shaping species health and the consideration of a full suite of processes, as well as their synergistic interplay, when considering the health of wildlife populations.

## Author Contributions

**Melanie R. Wells:** conceptualization (equal), data curation (equal), formal analysis (equal), funding acquisition (equal), investigation (equal), methodology (equal), project administration (equal), writing – original draft (equal), writing – review and editing (equal). **Annie Philips:** conceptualization (equal), funding acquisition (equal), writing – original draft (equal), writing – review and editing (equal). **Mary‐Anne Lea:** conceptualization (equal), funding acquisition (equal), writing – original draft (equal), writing – review and editing (equal). **Scott Carver:** conceptualization (equal), formal analysis (equal), funding acquisition (equal), project administration (equal), supervision (equal), writing – original draft (equal), writing – review and editing (equal).

## Conflicts of Interest

The authors declare no conflicts of interest.

## Supporting information


Appendix S1.

## Data Availability

Included on the title page: Data and code associated with this manuscript are archived in the Research Data Portal (RDP) of the University of Tasmania and can be accessed here: https://dx.doi.org/10.25959/jv3s‐5008 .
