# Supplementary material for: Spatial Variation in Body Condition of a Coastal Sentinel, the Little Penguin, Reflects Marine and Terrestrial Factors
Source: Ecol Evol. 2025 May 6;15(5):e71361. doi: 10.1002/ece3.71361 (PMC12055453; doi:10.1002/ece3.71361)
Supplement: Supplementary file 1 — Appendix S1. [file ECE3-15-e71361-s001.docx]

| SITE | Map location | Mean BC ± sem | Mean SST ± sem | Mean chla ± sem |
| --- | --- | --- | --- | --- |
| Bicheno | 5 | 9.89 ± 0.28 | 13.24 ± 0.00 | 1.00 ± 0.00 |
| Burnie | 12 | 8.53 ± 0.30 | 12.94 ± 0.00 | 0.76 ± 0.00 |
| Coswell | 4 | 10.38 ± 0.27 | 12.91 ± -0.02 | 1.88 ± -0.13 |
| Derwent | 1 | 9.73 ± 0.19 | 12.39 ± -0.01 | 5.64 ± -0.02 |
| Diamond Island | 6 | 9.78 ± 0.15 | 13.24 ± 0.00 | 1.00 ± 0.00 |
| Doctors Rocks | 13 | 8.75 ± 0.20 | 12.93 ± 0.00 | 0.79 ± 0.00 |
| Furneaux Islands | 7 | 8.79 ± 0.19 | 12.66 ± 0.00 | 0.78 ± 0.00 |
| King Island | 15 | 8.79 ± 0.23 | 13.54 ± 0.00 | 0.48 ± 0.00 |
| Lillico | 9 | 8.93 ± 0.48 | 13.52 ± 0.01 | 0.48 ± 0.00 |
| Little Christmas Island | 3 | 9.90 ± 0.28 | 12.89 ± 0.01 | 1.95 ± 0.00 |
| Low Head | 8 | 8.96 ± 0.19 | 12.91 ± 0.01 | 0.75 ± 0.00 |
| Pirates Bay | 2 | 9.37 ± 0.33 | 12.72 ± 0.02 | 1.53 ± 0.00 |
| Stanley | 14 | 8.97 ± 0.70 | 12.91 ± 0.00 | 2.21 ± 0.00 |
| Sulphur Creek | 11 | 9.42 ± 0.29 | 12.96 ± 0.00 | 0.91 ± 0.00 |
| Ulverstone | 10 | 8.82 ± 0.24 | 12.97 ± 0.00 | 0.89 ± 0.00 |

**Appendix S1**

Summary of study sites, body condition (BC, gmm^-1^) of breeding little penguins, sea surface temperature (SST) (°C) and chlorophyll-*a* (chla) (mgm⁻³) extracted from mean daily average from within 30km of site over the two-month period corresponding to timing of each sampling period. Data represented in mean and standard error of the mean (SEM).


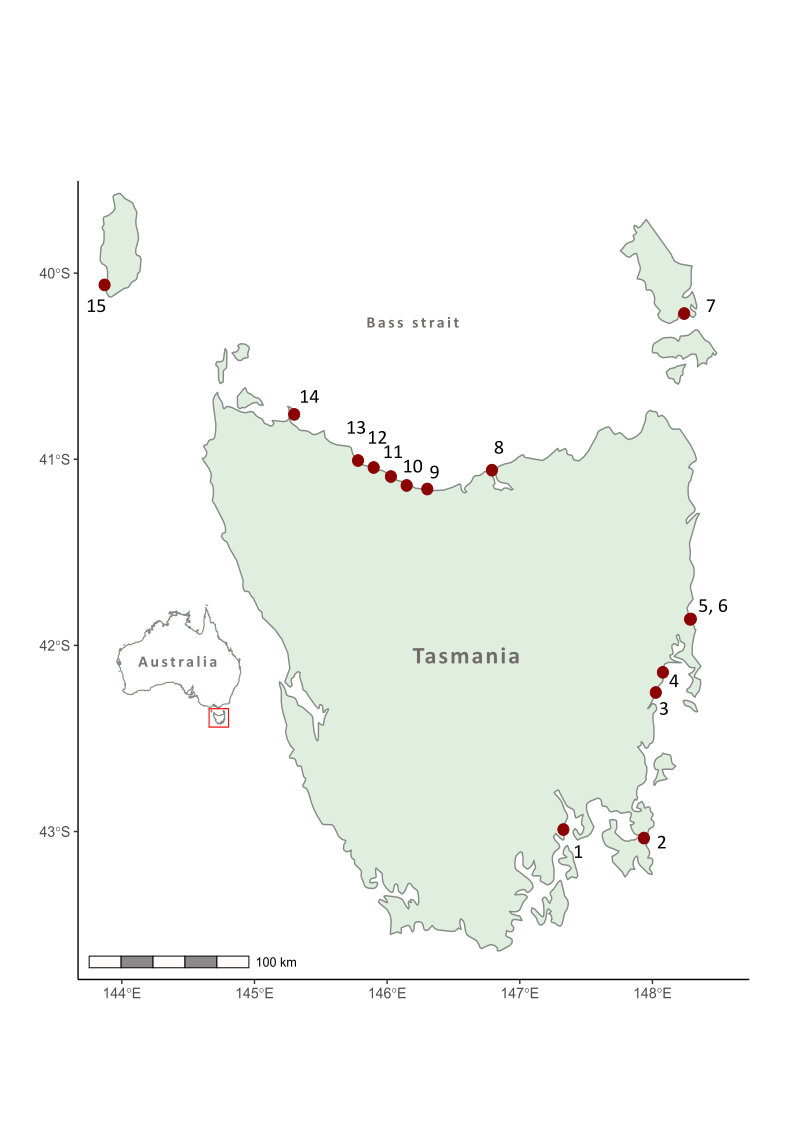


| Site | n (M, F) | Coast | Mean BC ± sem | Road length (km) | Industry |
| --- | --- | --- | --- | --- | --- |
| Bicheno | 4 (3,1) | east | 9.89 ± 0.28 | 5.66 | Y |
| Burnie | 14 (6,8) | north | 8.53 ± 0.30 | 27.53 | Y |
| Coswell | 10 (10,0) | east | 10.38 ± 0.27 | 6.14 | Y |
| Derwent | 15 (8,7) | east | 9.73 ± 0.19 | 22.52 | N |
| Diamond Island | 5 (2,3) | east | 9.78 ± 0.15 | 3.09 | Y |
| Doctors Rocks | 18 (13,5) | north | 8.75 ± 0.20 | 8.21 | Y |
| Furneaux Islands | 4 (4,0) | east | 8.79 ± 0.19 | 0 | N |
| King Island | 9 (5,4) | north | 8.79 ± 0.23 | 4.59 | N |
| Lillico | 9 (2,7) | north | 8.93 ± 0.48 | 9.25 | Y |
| Little Christmas Island | 14 (7,7) | east | 9.90 ± 0.28 | 0.86 | N |
| Low Head | 9 (6,3) | north | 8.96 ± 0.19 | 3.07 | Y |
| Pirates Bay | 8 (4,4) | east | 9.37 ± 0.33 | 9.87 | N |
| Stanley | 3 (0,5) | north | 8.97 ± 0.70 | 17.04 | Y |
| Sulphur Creek | 12 (7,8) | north | 9.42 ± 0.29 | 13.71 | Y |
| Ulverstone | 18 (13,5) | north | 8.82 ± 0.24 | 21.32 | Y |

**Appendix S1** (*cont.*)

Summary of study sites; sample size for males (M) and female (F); region along the coast; mean body condition (BC) ± standard error of the mean (sem); total road length around 1km of the site and the presence of industry within 10km of the colony.

**Appendix S2**

Assessment of posterior distributions generated from the brms model.


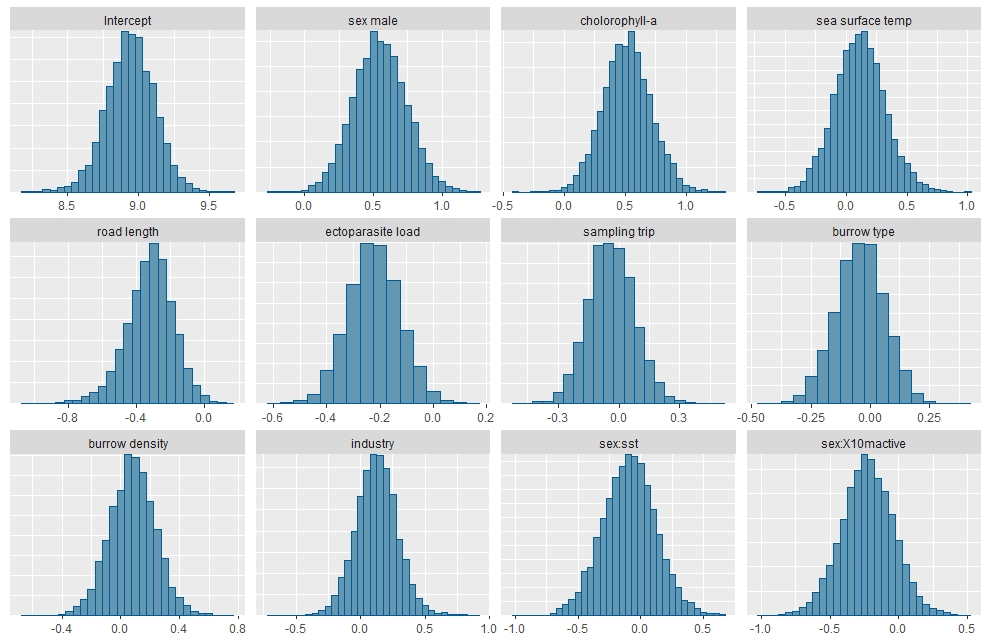


Assessment of model adequacy with 20,000 simulated (red) and observed (black) body condition data points from the posterior parameter estimates (using the posterior_predict function from Stan), including random noise, consistent with the residual standard deviations. Vertical bands depict 90% of the simulated values.


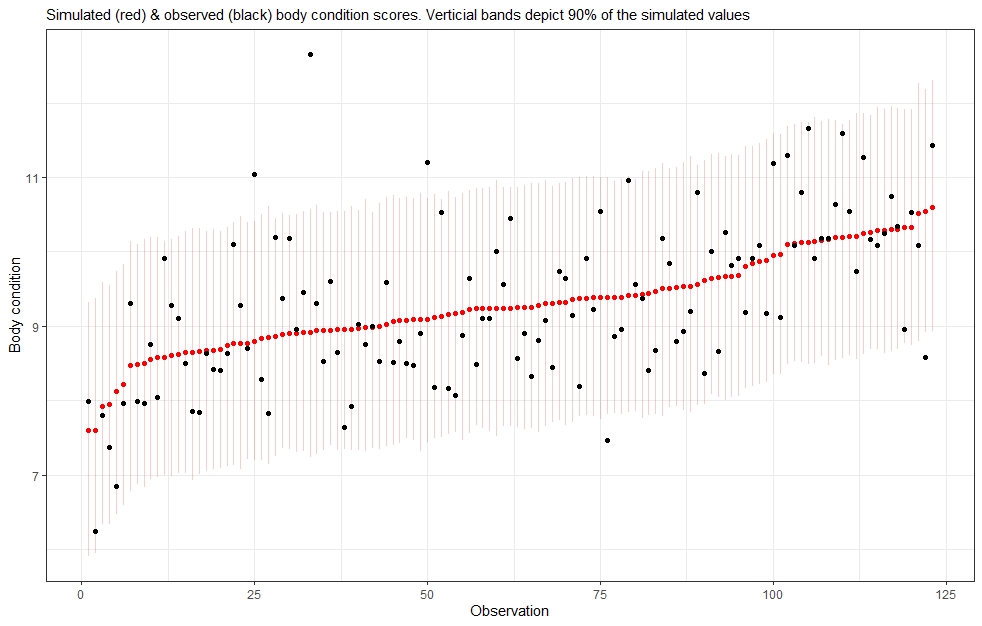


**Appendix S3**

Box plots of little penguin body condition (gmm^-1^) at breeding sites around Tasmania
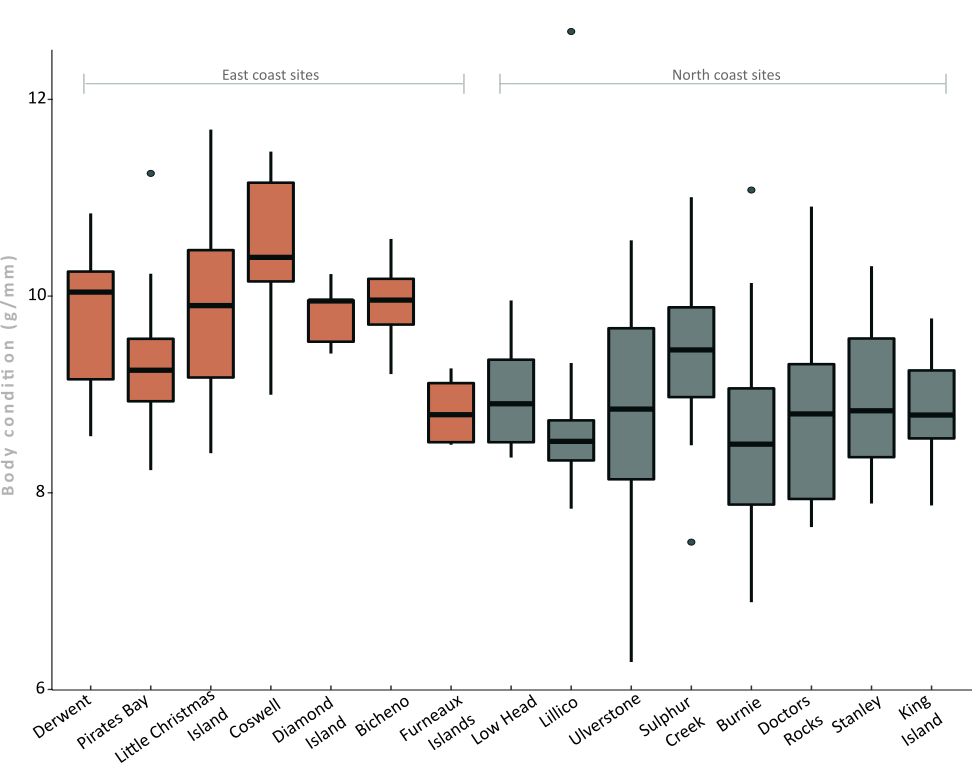


**Appendix S4**

Relationships of all other parameters used in the model with body condition. These parameters were defined as insignificant determinants of little penguin body condition based on the 95% credible intervals from the Bayesian multilevel model overlapping zero. Both burrow density and sea surface temperature were also included with sex interaction terms due to different trends between males and females. Black regression line is for the overall dataset (123 observations) with 95% confidence intervals. Males are coloured in orange with dotted regression line and females in blue.

**
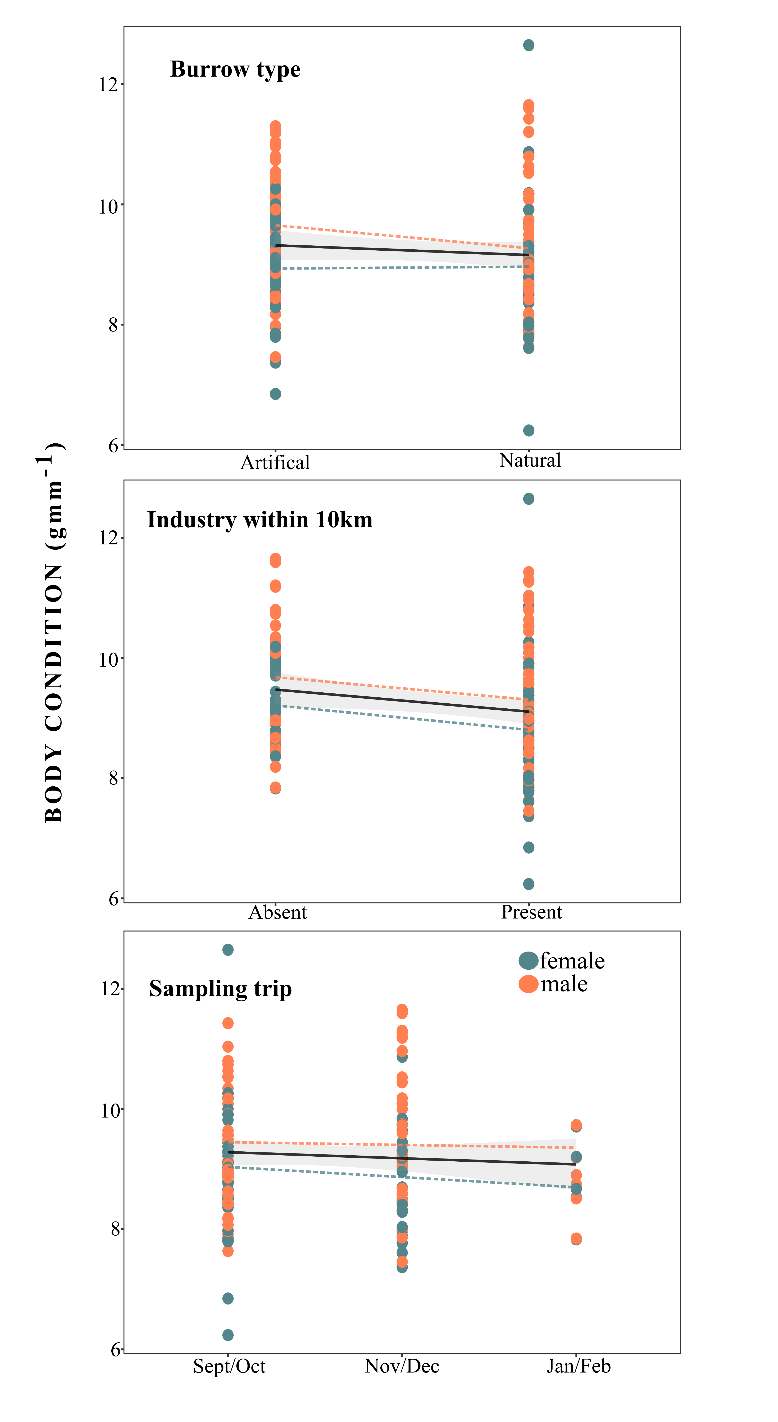
**

**
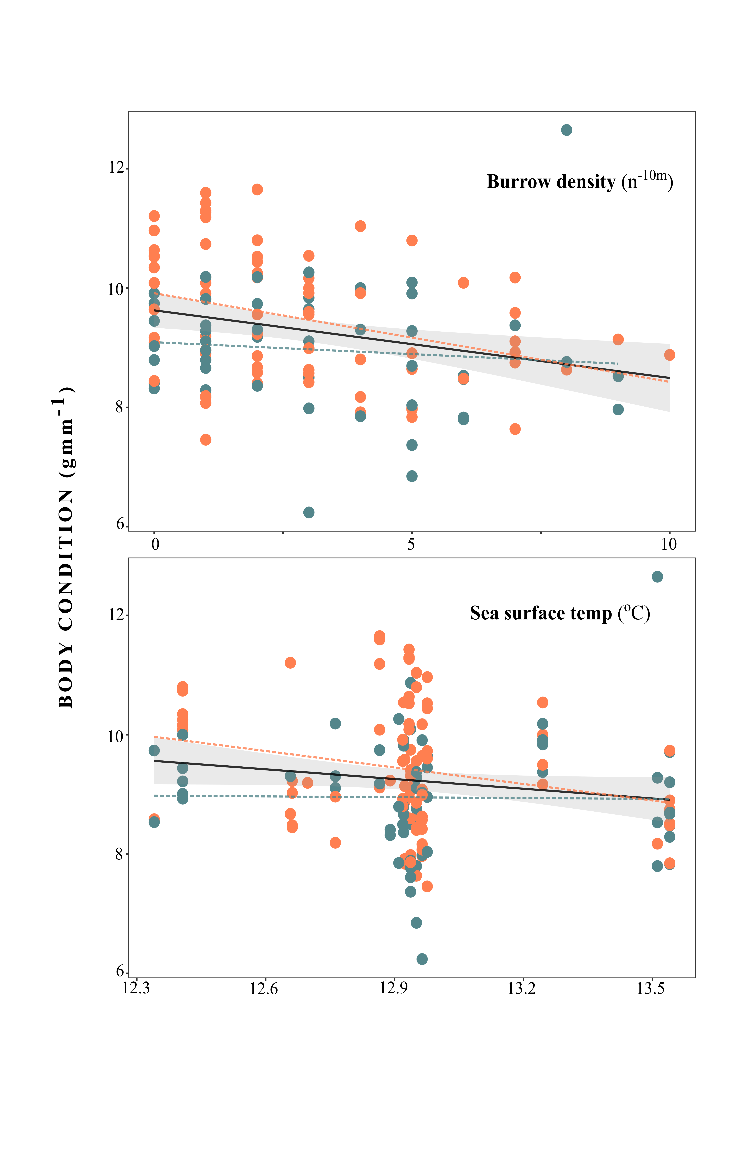
**

**Appendix S5**

Summary statistics for overall dataset for all morphometrics based on sex (mean ± SD). Sex was determined based on Bill Depth. All morphometric units are millimetres except for mass which is in grams.

Linear regression output and plot of ratio index of Mass by Flipper length for females and males separately.

|  | Est. | Std Error | t-value | p-value |
| --- | --- | --- | --- | --- |
| Intercept | -504.113 | 451.458 | -1.117 | 0.26606 |
| Flipper | 13.445 | 4.029 | 3.33 | 0.00108 |
| SexMale | 281.745 | 562.689 | 0.501 | 0.61736 |
| Flipper:SexMale | -2.102 | 4.961 | -0.424 | 0.67237 |

Residual standard error: 116.5 on 140 degrees of freedom

Multiple R-squared: 0.2533, Adjusted R-squared: 0.2373

F-statistic: 15.83 on 3 and 140 DF, p-value: 6.419e-09

| Sex | Mass | Flipper | Head Length | Bill Length | Bill Depth |
| --- | --- | --- | --- | --- | --- |
| Female | 1002 ± 125 | 112 ± 3.83 | 94.5 ± 3.02 | 37.4 ± 1.71 | 12.5 ± 0.47 |
| Male | 1092 ± 12 | 116 ± 4.36 | 98.4 ± 2.76 | 39.6 ± 1.79 | 14.3 ± 0.70 |

**
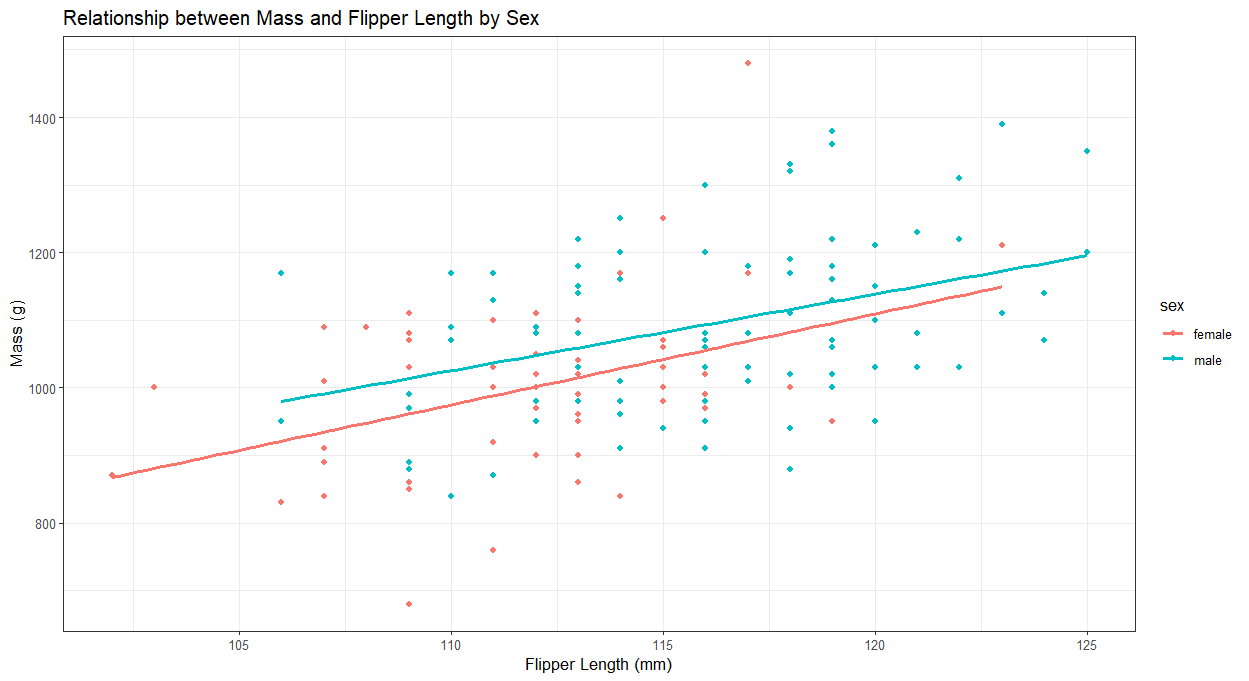
**
